# The Serum Expression of Selected miRNAs in Pulmonary Sarcoidosis with/without Löfgren's Syndrome

**DOI:** 10.1155/2016/1246129

**Published:** 2016-12-05

**Authors:** Eva Novosadova, Alzbeta Chabronova, Vitezslav Kolek, Martin Petrek, Zdenka Navratilova

**Affiliations:** ^1^Laboratory of Immunogenomics, Department of Pathological Physiology, Faculty of Medicine and Dentistry, Palacky University, Olomouc, Czech Republic; ^2^Department of Respiratory Medicine and TBC, Palacky University, Olomouc, Czech Republic; ^3^Institute of Molecular and Translational Medicine, Faculty of Medicine and Dentistry, Palacky University, Olomouc, Czech Republic

## Abstract

*Purpose*. Pulmonary sarcoidosis is associated with dysregulated expression of intracellular miRNAs. There is however only little information on extracellular miRNAs and their association with the disease course in sarcoidosis. We therefore assessed serum miRNAs in sarcoidosis classified according to the presence of Löfgren's syndrome (LS) as a hallmark of good prognosis in contrast to more advanced disease course.* Methods*. RT-PCR was used to assess 35 miRNAs in 13 healthy controls and 24 sarcoidosis patients (12 with X-ray (CXR) stage ≤ 1 and LS and 12 with insidious onset and CXR stage ≥ 3).* Results*. Compared to controls, we consistently observed dysregulated expressions of miR-146, miR-16, miR-425-5p, and miR-93-5p in both sarcoidosis groups irrespective of disease course. Specifically, patients without LS had dysregulated expressions of miR-150-5p, miR-1, and miR-212 compared to controls. Patients with LS had dysregulated expressions of miR-21-5p and miR-340-5p compared to controls. Bioinformatics predicted consistently “Pathways in cancer” to be modulated by both altered profiles in patients with/without LS. Three miRNAs (miR-21-5p, miR-340-5p, and miR-212-3p) differed between our patients with LS and those without LS; their cumulative effect may modulate “TGF-*β* signalling pathway.”* Conclusions*. Further study should focus on possible applications of serum miRNAs for diagnostics follow-up and for prognosis.

## 1. Introduction 

Pulmonary sarcoidosis is an inflammatory granulomatous disease of unknown cause(s) [1]. The course and prognosis of sarcoidosis vary greatly. High rate of spontaneous remissions is known in patients with early disease that present constellation of specific features called Löfgren's syndrome. On the other hand, an insidious onset of the disease without Löfgren's syndrome may be followed by progressive fibrosis [1].

Sarcoidosis progression has not been associated with any particularly specific immunological parameters although sarcoidosis inflammation is generally characterized by elevated production of proteolytic enzymes, cytokines and chemokine ligands/receptors, and other molecules with immune-regulatory functions [1, 2]. Expression of these proinflammatory factors is modulated by miRNome that composes of numerous small single-stranded (20–24 nucleotides long) noncoding RNAs termed miRNAs (alias microRNAs). They bind to complementary mRNA sequences within target genes whose expression is subsequently regulated through posttranscriptional repression [3]. The regulatory property of miRNAs is generally associated with their cytoplasmic accumulation in a cell [3].

In pulmonary sarcoidosis, the intracellular expression of miRNAs has been investigated in bronchoalveolar cells, peripheral blood mononuclear cells, and the lung tissue [4–6]. In addition, some intracellular miRNAs have been reported to have altered expression during sarcoidosis progression [4, 5].

Besides their intracellular accumulation, however, miRNAs are known to be present in extracellular fluids [7–9]. Little information on the extracellular miRNAs only exists in sarcoidosis [7]. In addition, the sparse data should be confirmed with regard to current recommendation for normalisation strategy of extracellular miRNAs [7, 10].

We have therefore investigated expression stability of serum miRNAs in sarcoidosis and then compared serum expression of miRNAs in sarcoidosis patients classified according to the presence of Löfgren's syndrome as a hallmark of good prognosis in comparison to group of patients with more advanced disease course.

## 2. Methods

### 2.1. Subjects

Serum samples were obtained from 13 healthy controls and 24 patients with pulmonary sarcoidosis according to a standard protocol [11]. Diagnosis of pulmonary sarcoidosis was made according to the criteria of ATS/ERS/WASOG International Consensus Statement [12]. Löfgren's syndrome was characterized by erythema nodosum, bilateral hilar lymphadenopathy, fever, and polyarthritis. All patients with Löfgren's syndrome had chest X-ray (CXR) stage ≤ 1 (*n* = 12) and all patients without Löfgren's syndrome had CXR stage ≥ 3 (*n* = 12). Clinical characteristics and bronchoalveolar cellular profile are provided in Tables 1 and 2, respectively.

All patients were recruited at the Department of Respiratory Medicine and TBC, University Hospital in Olomouc, the Czech Republic. The study was performed with the approval of Ethical committees of Medical Faculty PU & University Hospital, Olomouc. Informed consent for the anonymous usage of all serum samples for the purposes of the study was obtained from all enrolled subjects.

### 2.2. RNA Isolation and RT-PCR

To ensure sample quality without erythrocyte miRNAs contamination, the level of haemolysis in all serum samples was assessed by spectrophotometry (NanoDrop 1000, USA) [11]. No serum sample had A414 reading above a value of 0.2 [13].

Extracellular RNA was isolated from 300 *μ*L of serum by using miRCURY™ RNA isolation kit for biofluids (Exiqon, Denmark). PCR conditions and all reaction mixes were adopted from the user bulletin by Applied Biosystems (Protocol for Creating Custom RT and Preamplification Pools using TaqMan® MicroRNA Assays).

Briefly, multiplex reverse transcription (RT) was performed with TaqMan MicroRNA Reverse Transcription kit (Applied Biosystems, CA, USA) and RT primer pool prepared by mixing 5x RT primers provided in TaqMan MicroRNA Assays (Applied Biosystems, CA, USA). To preamplify all tested miRNAs together, TaqMan PreAmp Master Mix was used with PreAmp Primer Pool prepared from 20x TaqMan MicroRNA Assay (Applied Biosystems, CA, USA). qPCRBIO Probe Mix No-ROX (PCR Biosystems, United Kingdom) and 20x TaqMan MicroRNA Assays (Applied Biosystems, CA, USA) were used to perform RT-PCR of individual miRNAs (RotorGene3000 system Corbett Research, Sydney, Australia). All TaqMan MicroRNA Assays are listed in Supplementary material (Online Resource 1 in Supplementary Material available online at http://dx.doi.org/10.1155/2016/1246129).

### 2.3. Data Analysis and Statistics

Second derivative method, described previously in our laboratory [14], was used to assess Cq and efficiency of RT-PCR. The particular miRNAs were selected based on early published screening data in sarcoidosis [7, 15] and our pilot qPCR data that were partly published at European Respiratory Society Congress in 2015 [16]. Only miRNAs with efficiency within 1.7–2.0 (27 miRNAs) were utilised for the subsequent analyses [17] (Supplementary Material/Online Resource 1). GeNorm and NormFinder algorithms were used to reveal a normalisation factor with the highest stability [18, 19].

Regarding univariate statistical analysis, Mann–Whitney* U* test was used to detect possible differences in relative expressions of miRNAs between the study groups. To account for a high false-positive rate possibly caused by a multiple comparison among 3 study groups, correction of *p* value was performed by using false discovery rate (FDR) method according to Benjamini and Hochberg [20] where desired FDR equaled 5% and a *p* value ≤ 0.03 was considered to be significant.

Multivariate analysis was performed with SIMCA P version 13.5.0 (Umetrics, AB, Umeå, Sweden) by using principle component analysis (PCA) and orthogonal projections to latent structures (OPLS) analysis [21]. Analyses were performed on log⁡2-transformed, quantile-normalised, mean-centered data scaled to unit variance. Model performance is reported as cumulative correlation coefficient for the model (*R* ^2^), predictive performance based on 7-fold cross-validation (*Q* ^2^), and cross-validated ANOVA (CV-ANOVA) *p* values for OPLS-based group separation [22].

### 2.4. Pathway Analysis

MiRSystem (ver. 20150312) was used to assess the possible cumulative effect of the dysregulated miRNAs on gene expression in sarcoidosis [23]. The fold changes of means (patients/controls) of the altered miRNAs were used as an input, including* Kyoto Encyclopedia of Genes and Genomes* (KEGG) Pathways and an observed-to-expected ratio of greater than 1.0. To balance the reliability of the predictions with a manageable number of records, a number of algorithms predicting the same miRNA-gene interaction pair were set at three algorithm hits, including validated miRNA-gene pairs. Only biological functions/pathways with at least 25 genes and at most 500 genes were analysed.

## 3. Results

### 3.1. Univariate Analysis of miRNAs in Pulmonary Sarcoidosis

To increase reliability of qPCR data on extracellular miRNAs, several normalisation strategies were tested before own statistical analysis comparing possible differences among study groups. Briefly, both NormFinder and GeNorm algorithms consistently showed a geometric mean of 23 extracellular miRNAs that were expressed in all subjects, to have the lowest expression variability within all samples.

**Figure 1:**
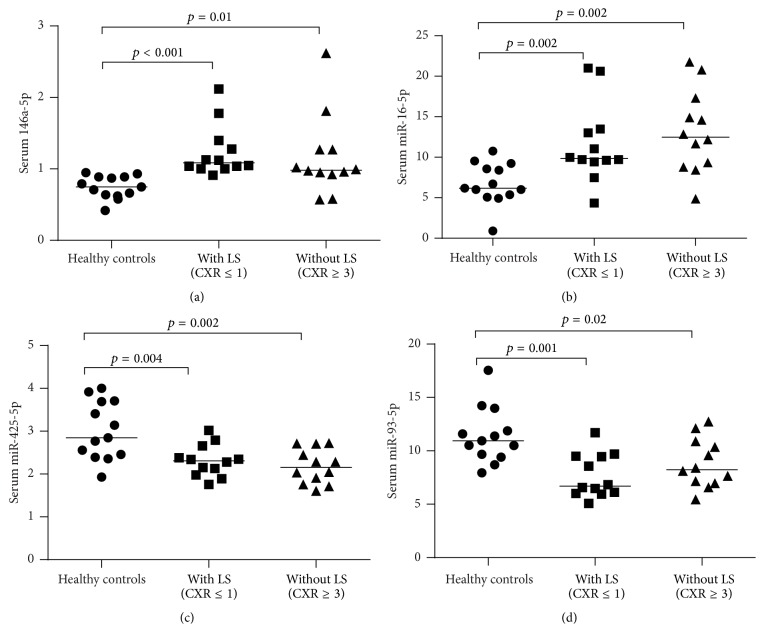
Consistently dysregulated serum miRNAs in both groups of the studied sarcoidosis patients with Löfgren's syndrome and without Löfgren's syndrome compared to healthy controls. CXR, chest X-ray and LS, Löfgren's syndrome.

**Figure 2:**
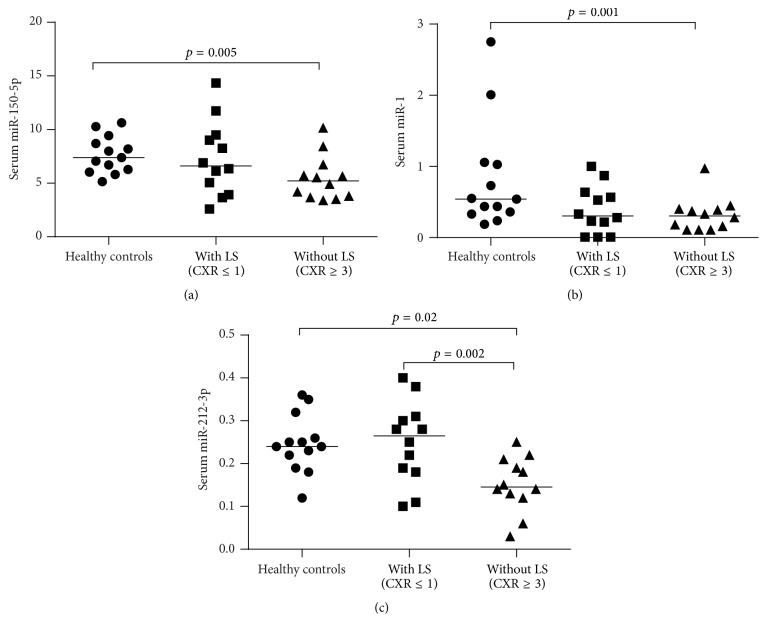
Serum miRNAs only dysregulated in sarcoidosis patients without Löfgren's syndrome compared to healthy controls. CXR, chest X-ray and LS, Löfgren's syndrome.

**Figure 3:**
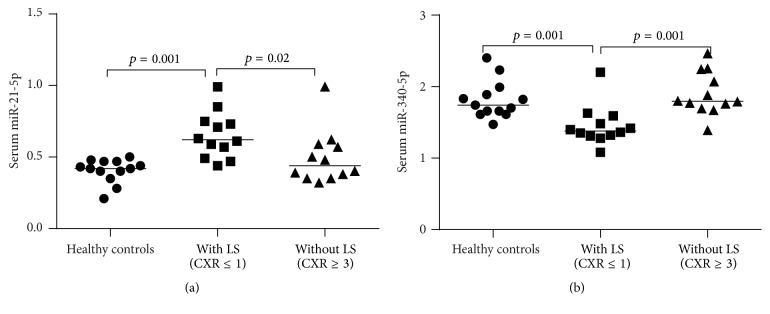
Serum miRNAs only dysregulated in our sarcoidosis patients with Löfgren's syndrome compared to healthy controls. CXR, chest X-ray and LS, Löfgren's syndrome.

In comparison to healthy controls, we consistently observed the increased expressions of miR-146a-5p and miR-16-5p and decreased expressions of miR-425-5p and miR-93-5p in both groups of our sarcoidosis patients with/without Löfgren's syndrome (Figures 1(a)–1(d)). Serum expressions of three miRNAs (miR-150-5p, miR-1, and miR-212-3p) were decreased in our patients without Löfgren's syndrome compared to healthy controls (Figures 2(a)–2(c)). Serum miR-21-5p was increased in our patients with Löfgren's syndrome compared to healthy controls. By contrast, miR-340-5p was decreased in the same patients with Löfgren's syndrome compared to healthy controls (Figures 3(a) and 3(b)). The patients without Löfgren's syndrome had decreased expressions of miR-212-3p and miR-21-5p and increased expression of miR-340-5p in comparison with those with Löfgren's syndrome (Figures 2(c), 3(a), and 3(b)).

### 3.2. Multivariate Analysis of miRNAs in Pulmonary Sarcoidosis

Multivariate analysis was performed to investigate an effect of coexpression of several dysregulated serum miRNAs in sarcoidosis (Supplementary Material/Online Resource 2). Both multivariate analyses of 6 and 7 miRNAs that were dysregulated either in the patients with Löfgren's syndrome or in the patients without Löfgren's syndrome showed consistently that OPLS modelling provides a significant separation between our patients with pulmonary sarcoidosis irrespective of disease course and the healthy controls resulting in the predictive power of 72% and 65% (*R* ^2^ = 0.737 and *R* ^2^ = 0.691702 and *Q* ^2^ = 0.717 and *Q* ^2^ = 0.652; *p* = 9.2 × 10^−7^ and *p* = 9.16 × 10^−6^) based on 7-fold cross-validation (Supplementary Material/Online Resource 2). The multivariate modelling with three miRNAs that were dysregulated in the univariate analysis between our patients with Löfgren's syndrome and those without Löfgren's syndrome showed a significant separation with a poor reproducibility in our training data set resulting in the poor predictive power of 44% (*R* ^2^ = 0.536 and *Q* ^2^ = 0.443; *p* = 2.2 × 10^−3^; Supplementary material/Online resource 2).

### 3.3. Pathway Analysis

To reveal cumulative effect of the dysregulated miRNAs on gene expression, pathway analysis with miRSystem database was performed. The “Pathways in Cancer” was consistently predicted to be targeted with the highest miRSystem score by both expression profiles that were dysregulated in the patients with/without Löfgren's syndrome compared to healthy controls (*p* = 5.0 × 10^−9^ and *p* = 9.0 × 10^−7^; Supplementary Material/Online Resource 3). In this pathway, all 6 miRNAs (miR-146a-5p, miR-16-5p, miR-425-5p, miR-425-5p, miR-21-5p, and miR-340-5p) that were dysregulated in the patients with Löfgren's syndrome were predicted to modulate 103 target genes based on experimental validation according miRSystem (Table 3). Further, all 7 miRNAs (miR-146a-5p, miR-16-5p, miR-425-5p, miR-425-5p, miR-150-5p, miR-1, and miR-212-3p) that were dysregulated in the patients without Löfgren's syndrome were predicted to modulate 112 target genes based on experimental validation according miRSystem (Table 3).

The “Transforming Growth Factor (TGF)-*β* signalling pathway” with its highest miRSystem score among KEGG pathways was predicted to be significantly affected (*p* = 1.9 × 10^−10^; Supplementary material/Online Resource 3) by the cumulative effect of three miRNAs whose serum expressions were dysregulated between our patients with Löfgren's syndrome and those without Löfgren's syndrome. In this pathway, three miRNAs (miR-340-5p, miR-212-3p, and miR-21-5p) targeted 25 experimentally validated genes (Table 3).

## 4. Discussion

This work attempts to provide an insight into differential expression of extracellular miRNAs in sarcoidosis patients classified according to the presence of Löfgren's syndrome as a hallmark of good prognosis in comparison to advanced disease course. Our geometric mean-normalised expression showed that serum miR-146a-5p, miR-16-5p, miR-425-5p, and miR-93-5p are consistently dysregulated, regardless of sarcoidosis prognosis, in our patients with pulmonary sarcoidosis compared to healthy controls. Specifically, patients without Löfgren's syndrome had dysregulated expressions of miR-150-5p, miR-1, and miR-212-3p and those with Löfgren's syndrome had dysregulated miR-21-5p and miR-340-5p in comparison to healthy controls. MiRSystem predicted the Pathways in cancer to be consistently affected by both of the dysregulated expression profiles in sarcoidosis with/without Löfgren's syndrome. Three serum miRNAs (miR-21-5p, miR-340-5p, and miR-212-3p) differed between the sarcoidosis patients with Löfgren's syndrome and those without Löfgren's syndrome. Their cumulative effect may modulate the “transforming growth factor (TGF)-*β* signalling pathway” during sarcoidosis progression.

Because of a lack of current knowledge on stably expressed extracellular miRNAs in serum from the patients with sarcoidosis, the best approach of qPCR data normalisation was investigated at the earliest. A geometric mean of all expressed extracellular miRNAs showed the best stability and was therefore used to normalise the raw qPCR data in subsequent analysis comparing the study groups. The geometric mean-normalisation is different from that used by Jazwa et al. who measured like our paper serum expressions of miR-16-5p, miR-146a-5p, and miR-150-5p in sarcoidosis patients [7]. They did not find any of these serum miRNAs to be dysregulated in pulmonary sarcoidosis. It is in contrast with our observations and could be explain by the different normalisation approach in combination with different representation of CXR stages among the sarcoidosis patients in the study by Jazwa et al. [7].

In comparison with our healthy controls, dysregulation of 4 miRNAs was consistently presented in sarcoidosis as a whole. However, we also observed several miRNAs to be associated with either the presence of Löfgren's syndrome or its absence. Through these differences, the “Pathways in Cancer” was consistently predicted to be modulated by cumulative effect of both of the serum expressions profiles in pulmonary sarcoidosis with/without Löfgren's syndrome. Remarkably, an increased risk of cancer is discussed in sarcoidosis patients and the presence of sarcoidosis granuloma has been reported in case studies on oncology patients [24–26]. This relationship has been also supported by other authors [27]. The dysregulation of serum miRNAs in our sarcoidosis patients may therefore result from the inflammation, apoptosis, and angiogenesis frequently accompanying various malignant processes [28–30] and it likely may not be directly related to presence/absence of Löfgren's syndrome in sarcoidosis.

In addition, several particular target genes of the “Pathways in Cancer” have been indeed indicated in wet laboratory to be dysregulated at their protein and/or mRNA level in sarcoidosis [31–36]. Among them, WNT (wingless and integrase-1)7A, catenin-beta, and transforming growth factor- (TGF-) *β* are concurrently involved into other signalling pathways, including the WNT and TGF-*β* signalling pathways [23]. Both of these signalling pathways were already once predicted to be modulated by cumulative effect of several intracellular miRNAs that are dysregulated in the peripheral blood lymphocytes and the lung tissue obtained from sarcoidosis patients [6].

In line with the intracellular miRNAs-based prediction [6], the “TGF-*β* signalling pathway” was predicted here to be affected by the extracellular serum miRNAs (miR-21-5p, miR-340-5p, and miR-212-3p) that differed between our patients with Löfgren's syndrome and those without Löfgren's syndrome. Taking into consideration a profibrotic character of TGF-*β* action [37], it is notable that the “TGF-*β* signalling pathway” was predicted here although only two patients with fibrotic changes (CXR stage IV) were enrolled in this study. Besides them, the other patients without Löfgren's syndrome had CXR stage III in this work. We may therefore speculate on an incipient fibrotic process that involves posttranscriptional regulation beginning before CXR stage IV. On the other hand, our patients with CXR stage ≥ 3 did not have dysregulated expression of miR-21 whose elevation has been associated with IPF [38].

Thus, comparison among all 5 CXR stages needs to be elucidated to gain a whole insight into the disease course ranging from invisible abnormality of the intrathoracic lymph nodes toward the lung parenchymal involvement and lung fibrosis in the most advanced sarcoidosis [1]. This could even reveal the key regulatory player leading to the disease remission that is known to be common (55–90%) in early disease (namely, in Löfgren's syndrome) whereas rare (10–20%) and absent in CXR stage III and IV, respectively.

Our multivariate analysis showed significant separation between our sarcoidosis patients and healthy controls. However, three miRNAs (miR-21-5p, miR-340-5p, and miR-212-3p) did not provide good model to separate our sarcoidosis patients according to the presence/absence of Löfgren's syndrome. The lack of profound differences between two groups of our patients with sarcoidosis is in line with current poor knowledge on any sufficiently sensitive and specific serum profile for the disease course [39]. This seems not to stand for genetic background as several genetic variants have been reported to be associated with Löfgren's syndrome [40–42].

It should be noted that some miRNAs with plausible relevance for sarcoidosis pathogenesis were not investigated and this could represent a limitation of our study. Our multiplex qPCR method did not also allow us to perform any high-throughput screening, although we assessed higher number of serum miRNA than a previous study on serum miRNAs in sarcoidosis [7]. Our selection was based on the current knowledge on intracellular miRNAs in pulmonary sarcoidosis [6, 15]. Taking into consideration different biological properties, for example, the broadly discussed processing during cell-cell communication, intracellular miRNAs are unlikely to be dysregulated in parallel with their intracellular expression [43]. Thus, a high-throughput screening for extracellular miRNAs may reveal new serum biomarkers of sarcoidosis and the disease prognosis.

In conclusion, we report several serum miRNAs to be associated with pulmonary sarcoidosis and also further differences between our sarcoidosis patients stratified according to the presence/absence of Löfgren's syndrome as a hallmark of good prognosis. In an attempt to link the serum miRNAs to certain biological processes using bioinformatics tools, the “Pathways in Cancer” was predicted to be related to pulmonary sarcoidosis as a whole whereas the “TGF-beta signalling pathway” was predicted to be related to the disease course. The complex interplay between the serum miRNAs and the predicted target genes of these signalling pathways remains the matter of future experimental investigation to gain detailed insight into the pathological mechanisms underlying the disease and its advancement.

## Funding

The work was supported by Grant Projects LO1304, CZ.1.07/2.3.00/30.0004, and IGA PU LF 2015_030, 2016_009.

## Supplementary Material

Online Resource 1: A list of TaqMan assays. ID numbers of TaqMan MicroRNA Assays (Applied Biosystems, CA, USA) are listed with their miRNA sequences.
Online Resource 2: Our OPLS modelling showed significant separation between our sarcoidosis patients and healthy controls. Three miRNAs (miR-21, miR-340 and miR-212) however did not provide a good model to separate our sarcoidosis patients according to the presence/absence of Löfgren's syndrome.
Online Resource 3: The “Pathways in cancer” was consistently predicted to be affected by both expression profiles that were dysregulated in our patients with/without Löfgren's syndrome (LS) compared to healthy controls. The “TGF-beta signalling pathway” was predicted to be affected by the cumulative effect of three miRNAs whose serum expressions were dysregulated between our patients with LS and those without LS.

## Figures and Tables

**Table 1:** Clinical characteristics of our patients with pulmonary sarcoidosis and healthy controls.

	Healthy controls *N* = 13	Sarcoidosis	
		With LS (CXR ≤ 1) *N* = 12	Without LS (CXR ≥ 3) *N* = 12
Gender, men/women	2/11	8/4	6/6
Mean age (min–max)	45.6 (23–62)	45.5 (32–62)	46.6 (26–72)
Smoking history (nonsmoker/ex-smoker^*∗*^/current smoker/NA)	13/0/0/0	9/1/1/1	9/2/1/0
CXR stage 0/1	NA	12	NA
CXR stage 3/4	NA	NA	10/2
FEV1 (min–max) (%)	NA	106 (99–109)	90 (59–113)
FEV1/VC (min–max) (%)	NA	81 (69–90)	76 (74–80)
Mean DLCO (min–max) (%)	NA	101 (82–131)	78 (46–98)
Mean DLCO/VA (min–max) (%)	NA	90 (84–94)	103 (95–115)

NA, not available or not applicable; CXR, chest X-ray; LS, Löfgren's syndrome; DLCO, diffusing capacity of the lung for carbon monoxide; VA, alveolar volume and *∗* a nonsmoker was defined as a never smoker and an ex-smoker was defined as someone who has not been smoking for at least 2 years before serum collection. FEV1, forced expiratory volume in 1 second; VC, vital capacity.

**Table 2:** Bronchoalveolar cellular profile in our patients with pulmonary sarcoidosis.

Sarcoidosis	With LS (CXR ≤ 1) *N* = 12	Without LS (CXR ≥ 3) *N* = 12
Cellular profile	Mean (min–max)	Mean (min–max)
Total cell count 10^6^/mL	0.85 (0.40–1.35)	0.72 (0.10–1.40)
Macrophage absolute count	0.66 (0.28–1.02)	0.58 (0.09–1.40)
Macrophage relative count (%)	77.31 (57.00–91.10)	76.67 (43.00–90.70)
Lymphocytes absolute count	0.18 (0.06–0.45)	0.14 (0.01–0.33)
Lymphocytes relative count (%)	20.58 (8.60–41.00)	20.08 (8.00–46.00)
Neutrophils absolute count	0.01 (0.00–0.04)	0.02 (0.00–0.05)
Neutrophils relative count (%)	1.68 (0.00–8.00)	2.27 (0.00–6.00)
Eosinophils absolute count	0.00 (0.00–0.01)	0.00 (0.00–0.02)
Eosinophils relative count (%)	0.43 (0.00–2.00)	0.99 (0.00–7.00)
CD3^+^ absolute count	0.56 (0.00–1.13)	0.35 (0.00–1.06)
CD3^+^ relative count (%)	84.62 (58.00–94.00)	75.00 (36.00–96.00)
CD4^+^ absolute count	0.26 (0.00–0.79)	0.15 (0.00–0.67)
CD4^+^ relative count (%)	71.69 (35.00–90.00)	55.58 (29.00–89.00)
CD8^+^ absolute count	0.06 (0.00–0.26)	0.04 (0.00–0.19)
CD8^+^ relative count (%)	13.08 (3.00–31.00)	21.00 (4.00–53.00)
CD19^+^ absolute count	0.00 (0.00–0.01)	0.00 (0.00–0.02)
CD19^+^ relative count (%)	0.54 (0.00–2.00)	1.08 (0.00–2.00)
CD4^+^/CD8^+^	9.45 (1.13–30.00)	5.34 (0.55–22.25)

CXR, chest X-ray; LS, Löfgren's syndrome.

**Table 3:** The target genes that are predicted to be modulated by the dysregulated miRNAs in our patients with sarcoidosis according to miRSystem.

Target gene	Validation	Gene description	Number of miRNA		
			Healthy control versus LS	Healthy controls versus CXR III-IV	LS versus CXR III-IV
ACVR1	V	Activin A receptor, type I	0	0	1
ACVR1C	V	Activin A receptor, type IC	0	0	1
ACVR2A	V	Activin A receptor, type IIA	0	0	1
ACVR2B	V	Activin A receptor, type IIB	0	0	3
AKT3	V	v-akt murine thymoma viral oncogene homolog 3	2	3	0
APC	V	Adenomatous polyposis coli	0	1	0
APPL1	V	Adaptor protein, phosphotyrosine interaction, PH domain and leucine zipper containing 1	2	1	0
ARNT	V	Aryl hydrocarbon receptor nuclear translocator	1	3	0
AXIN2	V	Axin 2	1	1	0
BCL2	V	B-cell CLL/lymphoma 2	3	3	0
BCR	V	Breakpoint cluster region	2	2	0
BMPR2	V	Bone morphogenetic protein receptor, type II (serine/threonine kinase)	0	0	2
BRCA2	V	Breast cancer 2, early onset	1	1	0
CASP8	V	Caspase 8, apoptosis-related cysteine peptidase	1	1	0
CBL	V	Cbl proto-oncogene, E3 ubiquitin protein ligase	1	3	0
CCDC6	V	Coiled-coil domain containing 6	2	2	0
CCND1	V	Cyclin D1	2	3	0
CCNE1	V	Cyclin E1	1	2	0
CDC42	V	Cell division cycle 42	1	2	0
CDK6	V	Cyclin-dependent kinase 6	4	4	0
CDKN1A	V	Cyclin-dependent kinase inhibitor 1A (p21, Cip1)	2	2	0
COL4A1	V	Collagen, type IV, alpha 1	2	1	0
COL4A4	V	Collagen, type IV, alpha 4		1	0
CRK	V	v-crk avian sarcoma virus CT10 oncogene homolog	1	3	0
CRKL	V	v-crk avian sarcoma virus CT10 oncogene homolog-like	1	1	0
CTBP2	V	C-terminal binding protein 2	0	1	0
CTNNB1	V	Catenin (cadherin-associated protein), beta 1, 88 kDa	1	1	0
CUL2	V	Cullin 2	1	1	0
CYCS	V	Cytochrome c, somatic	1	1	0
DVL1	V	Dishevelled segment polarity protein 1	1	1	0
E2F1	V	E2F transcription factor 1	2	1	0
E2F2	V	E2F transcription factor 2	1	1	0
E2F3	V	E2F transcription factor 3	3	3	0
E2F5	V	E2F transcription factor 5, p130-binding	0	0	1
EGLN1	V	egl-9 family hypoxia-inducible factor 1	1	1	0
EGLN2	V	egl-9 family hypoxia-inducible factor 2	1	1	0
EGLN3	V	egl-9 family hypoxia-inducible factor 3	1	1	0
EP300	V	E1A binding protein p300		2	1
EPAS1	V	endothelial PAS domain protein 1	2	1	0
ETS1	V	v-ets avian erythroblastosis virus E26 oncogene homolog 1	0	1	0
FADD	V	Fas (TNFRSF6)-associated via death domain	1	1	0
FAS	V	Fas cell surface death receptor	2	1	0
FASLG	V	Fas ligand (TNF superfamily, member 6)	1	0	0
FGF1	V	Fibroblast growth factor 1 (acidic)	1	0	0
FGF13	V	Fibroblast growth factor 13	1	0	0
FGF14	V	fibroblast growth Factor 14	0	1	0
FGF2	V	Fibroblast growth factor 2 (basic)	1	1	0
FGF4	V	Fibroblast growth factor 4	1	1	0
FGF7	V	Fibroblast growth factor 7	3	3	0
FGF9	V	Fibroblast growth factor 9	1	1	0
FGFR1	V	Fibroblast growth factor receptor 1	1	1	0
FGFR2	V	Fibroblast growth factor receptor 2	1	1	0
FIGF	V	c-fos induced growth factor (vascular endothelial growth factor D)	1	1	0
FLT3	V	fms-related tyrosine kinase 3	1	1	0
FN1	V	Fibronectin 1		1	0
FOXO1	V	Forkhead box O1	1	1	0
FZD1	V	Frizzled family receptor 1	1	1	0
FZD10	V	Frizzled family receptor 10	1	1	0
FZD4	V	Frizzled family receptor 4	0	1	0
FZD7	V	Frizzled family receptor 7	0	1	0
GDF5	V	Growth differentiation factor 5	0	0	1
GRB2	V	Growth factor receptor-bound protein 2	1	1	0
HHIP	V	Hedgehog interacting protein	0	1	0
HIF1A	V	Hypoxia inducible factor 1, alpha subunit (basic helix-loop-helix transcription factor)	1	1	0
HSP90B1	V	Heat shock protein 90 kDa beta (Grp94), member 1	0	2	0
CHUK	V	Conserved helix-loop-helix ubiquitous kinase	1	1	0
IGF1	V	Insulin-like growth factor 1 (somatomedin C)	2	2	0
IGF1R	V	Insulin-like growth factor 1 receptor	1	1	0
IKBKB	V	Inhibitor of kappa light polypeptide gene enhancer in B-cells, kinase beta	1	2	0
IL8	V	Interleukin 8	1	1	0
ITGA2	V	Integrin, alpha 2 (CD49B, alpha 2 subunit of VLA-2 receptor)	1	1	0
ITGA3	V	Integrin, alpha 3 (antigen CD49C, alpha 3 subunit of VLA-3 receptor)	0	1	0
ITGA6	V	Integrin, alpha 6	1	0	0
ITGAV	V	Integrin, alpha V	1	0	0
JAK1	V	Janus kinase 1	2	1	0
JUN	V	Jun proto-oncogene	1	1	0
KRAS	V	Kirsten rat sarcoma viral oncogene homolog	1	3	0
LAMA3	V	Laminin, alpha 3	1	1	0
LAMC1	V	Laminin, gamma 1 (formerly LAMB2)	2	2	0
LAMC2	V	Laminin, gamma 2	0	2	0
LTBP1	V	Latent transforming growth factor beta binding protein 1	0	0	1
MAP2K1	V	Mitogen-activated protein kinase kinase 1	1	1	0
MAPK1	V	Mitogen-activated protein kinase 1	1	3	1
MAPK10	V	Mitogen-activated protein kinase 10	1	0	0
MAPK3	V	Mitogen-activated protein kinase 3	0	1	0
MAPK9	V	Mitogen-activated protein kinase 9	2	3	0
MET	V	Met proto-oncogene	0	1	0
MITF	V	Microphthalmia-associated transcription factor	1	0	0
MMP2	V	Matrix metallopeptidase 2 (gelatinase A, 72 kDa gelatinase, 72 kDa type IV collagenase)	1	1	0
MSH2	V	mutS homolog 2	2	1	0
NRAS	V	Neuroblastoma RAS viral (v-ras) oncogene homolog	1	1	0
PDGFA	V	Platelet-derived growth factor alpha polypeptide	0	1	0
PDGFRA	V	Platelet-derived growth factor receptor, alpha polypeptide	2	1	0
PIAS1	V	Protein inhibitor of activated STAT, 1	1	1	0
PIAS3	V	Protein inhibitor of activated STAT, 3	0	1	0
PIK3R1	V	Phosphoinositide-3-kinase, regulatory subunit 1 (alpha)	3	4	0
PIK3R3	V	Phosphoinositide-3-kinase, regulatory subunit 3 (gamma)	1	2	0
PITX2	V	Paired-like homeodomain 2	0	0	1
PPP2CB	V	Protein phosphatase 2, catalytic subunit, beta isozyme	0	0	1
PRKCA	V	Protein kinase C, alpha	1	1	0
PTEN	V	Phosphatase and tensin homolog	2	1	0
PTCH1	V	Patched 1	1	2	0
RAC1	V	Ras-related C3 botulinum toxin substrate 1 (rho family, small GTP binding protein Rac1)	1	0	0
RAF1	V	v-raf-1 murine leukemia viral oncogene homolog 1	1	1	0
RALA	V	v-ral simian leukemia viral oncogene homolog A (ras related)	0	1	0
RALBP1	V	ralA binding protein 1	2	1	0
RARB	V	Retinoic acid receptor, beta	2	3	0
RASSF5	V	Ras association (RalGDS/AF-6) domain family member 5	1	1	0
RB1	V	Retinoblastoma 1	1	2	0
RET	V	Ret proto-oncogene	1	1	0
RHOA	V	Ras homolog family member A	1	0	1
ROCK1	V	Rho-associated, coiled-coil containing protein kinase 1	0	0	1
RPS6KB1	V	Ribosomal protein S6 kinase, 70 kDa, polypeptide 1	0	0	1
RUNX1	V	Runt-related transcription factor 1	1	2	0
RUNX1T1	V	Runt-related transcription factor 1; translocated to, 1 (cyclin D-related)	3	2	0
SLC2A1	V	Solute carrier family 2 (facilitated glucose transporter), member 1	0	2	0
SMAD2	V	SMAD family member 2	2	2	2
SMAD3	V	SMAD family member 3	1	1	0
SMAD4	V	SMAD family member 4	1	1	0
SMAD5	V	SMAD family member 5	0	0	1
SMAD7	V	SMAD family member 7	0	0	1
SMURF1	V	SMAD specific E3 ubiquitin protein ligase 1	0	0	1
STAT1	V	Signal transducer and activator of transcription 1, 91 kDa	1	1	0
STAT3	V	Signal transducer and activator of transcription 3 (acute-phase response factor)	2	1	0
STK4	V	Serine/threonine kinase 4		1	0
TCF7	V	Transcription factor 7 (T-cell specific, HMG-box)	1	1	0
TCF7L1	V	Transcription factor 7-like 1 (T-cell specific, HMG-box)	2	2	0
TCF7L2	V	Transcription factor 7-like 2 (T-cell specific, HMG-box)	0	1	0
TFDP1	V	Transcription factor Dp-1	0	0	1
TGFB1	V	Transforming growth factor, beta 1	1	0	1
TGFBR1	V	Transforming growth factor, beta receptor 1	1	0	1
TGFBR2	V	Transforming growth factor, beta receptor II (70/80 kDa)	2	1	1
THBS1	V	Thrombospondin 1	0	0	1
TPM3	V	Tropomyosin 3	1	2	0
TRAF6	V	TNF receptor-associated factor 6, E3 ubiquitin protein ligase	1	1	0
VEGFA	V	Vascular endothelial growth factor A	2	3	0
VHL	V	Von Hippel-Lindau tumor suppressor, E3 ubiquitin protein ligase	1	1	0
WNT11	V	Wingless-type MMTV integration site family, member 11	1	0	0
WNT3A	V	Wingless-type MMTV integration site family, member 3A	1	1	0
WNT7A	V	Wingless-type MMTV integration site family, member 7A	1	1	0
XIAP	V	X-linked inhibitor of apoptosis	1	1	0
ZFYVE16	V	Zinc finger, FYVE domain containing 16	0	0	2

CXR, chest X-ray; LS, Löfgren's syndrome; V, min. one miRNA has experimental validation in miRSystem; 0, none of the dysregulated miRNAs.
