# Supplementary material for: The Serum Expression of Selected miRNAs in Pulmonary Sarcoidosis with/without Löfgren's Syndrome
Source: Mediators Inflamm. 2016 Dec 5;2016:1246129. doi: 10.1155/2016/1246129 (PMC5165170; doi:10.1155/2016/1246129)
Supplement: Supplementary file 1 — Online Resource 1: A list of TaqMan assays. ID numbers of TaqMan MicroRNA Assays (Applied Biosystems, CA, USA) are listed with their miRNA sequences. Online Resource 2: Our OPLS modelling showed significant separation between our sarcoidosis patients and healthy controls. Three miRNAs (miR-21, miR-340 and miR-212) however did not provide a good model to separate our sarcoidosis patients according to the presence/absence of Löfgren's syndrome. Online Resource 3: The “Pathways in cancer” was consistently predicted to be affected by both expression profiles that were dysregulated in our patients with/without Löfgren's syndrome (LS) compared to healthy controls. The “TGF-beta signalling pathway” was predicted to be affected by the cumulative effect of three miRNAs whose serum expressions were dysregulated between our patients with LS and those without LS. [file 1246129.f1.zip › supplementary material_1.pdf]

miRNA name	miRNA ID	miRNA sequence
let-7a-5p	ID 000377	UGAGGUAGUAGGUUGUAUAGUU
miR-1	ID 002222	UGGAAUGUAAAGAAGUAUGUAU
miR-16-5p	ID 000391	UAGCAGCACGUAAAUAUUGGCG
miR-17-5p	ID 002308	CAAAGUGCUUACAGUGCAGGUAG
miR-19a-3p	ID 000395	UGUGCAAAUCUAUGCAAAACUGA
miR-19b-3p	ID 000396	UGUGCAAAUCCAUGCAAAACUGA
miR-27a-5p	ID 002445	AGGGCUUAGCUGCUUGUGAGCA
miR-29b-3p	ID 000413	UAGCACCAUUUGAAAUCAGUGUU
miR-30b-5p	ID 000602	UGUAAACAUCCUACACUCAGCU
miR-31-5p•	ID 002279	AGGCAAGAUGCUGGCAUAGCU
miR-33b-3p•	ID 241007	CAGUGCCUCGGCAGUGCAGCCC
miR-92b-5p•	ID 002343	AGGGACGGGACGCGGUGCAGUG
miR-93-5p	ID 001090	CAAAGUGCUGUUCGUGCAGGUAG
miR-122a-5p	ID 002245	UGGAGUGUGACAAUGGUGUUUG
miR-125a-5p•	ID 002198	UCCCUGAGACCCUUUAACCUGUGA
miR-142-5p•	ID 002248	CAUAAAGUAGAAAGCACUACU
miR-142-3p	ID 000464	UGUAGUGUUUCCUACUUUAUGGA
miR-148a-3p	ID 000470	UCAGUGCACUACAGAACUUUGU
miR-155	ID 000479	UUAAUGCUAAUCGUGAUAGGGG
miR-181b-1*-3p	ID 462578	CUCACUGAACAAUGAAUGCAA
miR-191-5p	ID 002299	CAACGGAAUCCCAAAAGCAGCUG
miR-193a-3p	ID 002250	AACUGGCCUACAAAGUCCCAGU
miR-200b-5p	ID 002274	CAUCUUACUGGGCAGCAUUGGA
miR-204-5p	ID 000508	UUCCCUUUGUCAUCCUAUGCCU
miR-206•	ID 000510	UGGAAUGUAAGGAAGUGUGUGG
miR-212-3p	ID 000515	UAACAGUCUCCAGUCACGGCC
miR-214 -3p	ID 002306	ACAGCAGGCACAGACAGGCAGU
miR-302c-3p	ID 000533	UAAGUGCUUCCAUGUUUCAGUGG
miR-335-5p	ID 000546	UCAAGAGCAAUAACGAAAAAUGU
miR-340-5p	ID 2258	UUAUAAAGCAAUGAGACUGAUU
miR-362-3p•	ID 002117	AACACACCUAUUCAAGGAUUCA
miR-425-5p	ID 001516	AAUGACACGAUCACUCCCGUUGA
miR-451a	ID 001141	AAACCGUUACCAUUACUGAGUU
miR-487b-3p•	ID 1285	AAUCGUACAGGGUCAUCCACUU
miR-502-5p	ID 001109	AUCCUUGCUAUCUGGGUGCUA
miR-505*-5p	ID 002087	GGGAGCCAGGAAGUAUUGAUGU
miR-770-5p	ID 002002	UCCAGUACCACGUGUCAGGGCCA
miR-21-5p	ID 000397	UAGCUUAUCAGACUGAUGUUGA
miR-26a-5p	ID 000405	UUCAAGUAAUCCAGGAUAGGCU
miR-146a-5p	ID 000468	UGAGAACUGAAUUCCAUGGGUU
miR-150-5p	ID 000473	UCUCCCAACCCUUGUACCAGUG

Online Resource 1: A list of TaqMan assays

Legend: • These miRNAs were not analysed because of low PCR efficiency
