# Supplementary material for: The Serum Expression of Selected miRNAs in Pulmonary Sarcoidosis with/without Löfgren's Syndrome
Source: Mediators Inflamm. 2016 Dec 5;2016:1246129. doi: 10.1155/2016/1246129 (PMC5165170; doi:10.1155/2016/1246129)
Supplement: Supplementary file 1 — Online Resource 1: A list of TaqMan assays. ID numbers of TaqMan MicroRNA Assays (Applied Biosystems, CA, USA) are listed with their miRNA sequences. Online Resource 2: Our OPLS modelling showed significant separation between our sarcoidosis patients and healthy controls. Three miRNAs (miR-21, miR-340 and miR-212) however did not provide a good model to separate our sarcoidosis patients according to the presence/absence of Löfgren's syndrome. Online Resource 3: The “Pathways in cancer” was consistently predicted to be affected by both expression profiles that were dysregulated in our patients with/without Löfgren's syndrome (LS) compared to healthy controls. The “TGF-beta signalling pathway” was predicted to be affected by the cumulative effect of three miRNAs whose serum expressions were dysregulated between our patients with LS and those without LS. [file 1246129.f1.zip › supplementary material_2.pdf]

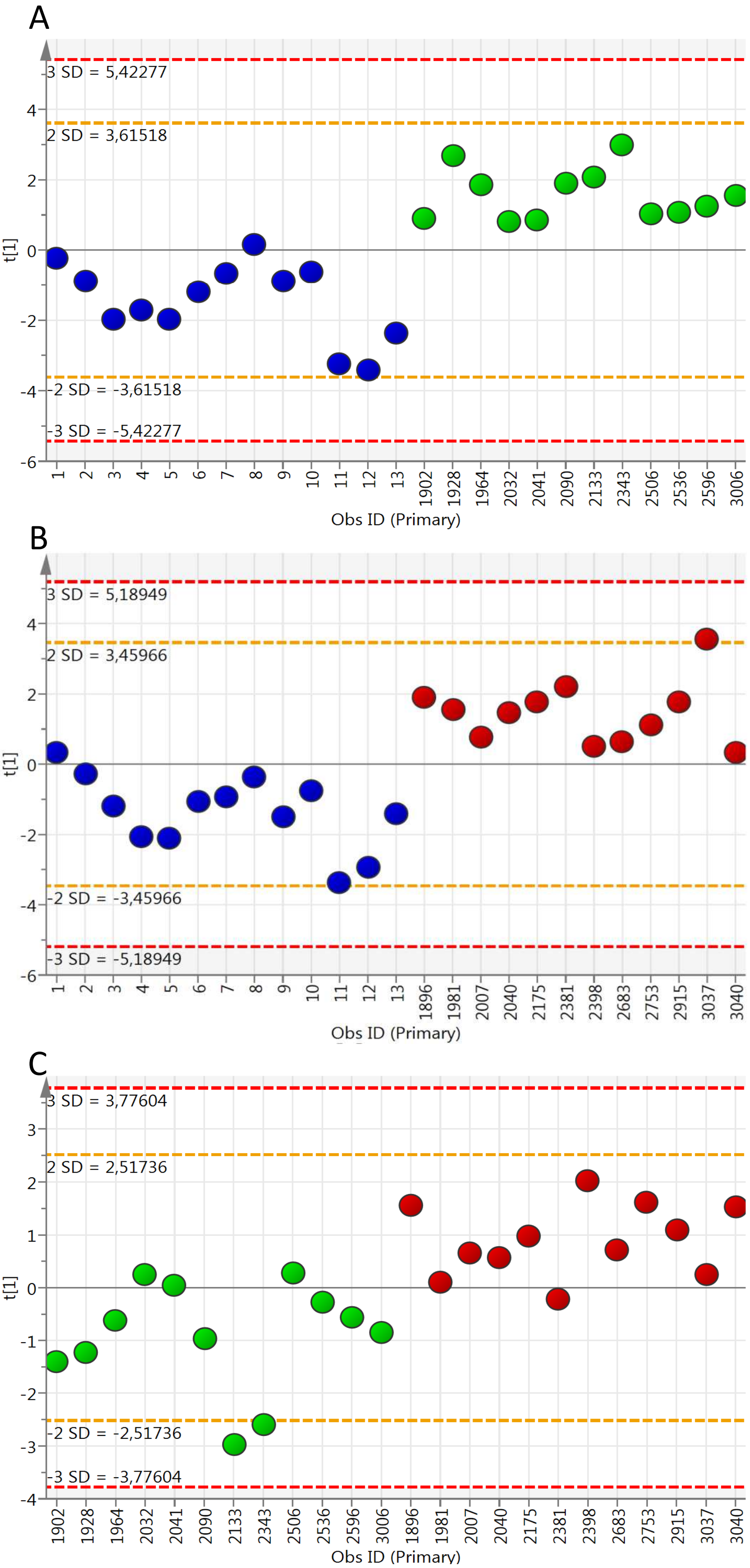

Online resource 2: Our OPLS modelling showed significant separation between our sarcoidosis patients and healthy controls (A: healthy controls vs Löfgren‘s syndrome and B: healthy controls vs an insidious onset of pulmonary sarcoidosis). Three miRNAs (c: miR-21, miR-340 and miR-212) did not provide a good model to separate our sarcoidosis patients according to the presence/absence of Löfgren‘s syndrome. (blue, healthy controls; green, Löfgren‘s syndrome and red, an insidious onset of pulmonary sarcoidosis)
