# Supplementary material for: The Serum Expression of Selected miRNAs in Pulmonary Sarcoidosis with/without Löfgren's Syndrome
Source: Mediators Inflamm. 2016 Dec 5;2016:1246129. doi: 10.1155/2016/1246129 (PMC5165170; doi:10.1155/2016/1246129)
Supplement: Supplementary file 1 — Online Resource 1: A list of TaqMan assays. ID numbers of TaqMan MicroRNA Assays (Applied Biosystems, CA, USA) are listed with their miRNA sequences. Online Resource 2: Our OPLS modelling showed significant separation between our sarcoidosis patients and healthy controls. Three miRNAs (miR-21, miR-340 and miR-212) however did not provide a good model to separate our sarcoidosis patients according to the presence/absence of Löfgren's syndrome. Online Resource 3: The “Pathways in cancer” was consistently predicted to be affected by both expression profiles that were dysregulated in our patients with/without Löfgren's syndrome (LS) compared to healthy controls. The “TGF-beta signalling pathway” was predicted to be affected by the cumulative effect of three miRNAs whose serum expressions were dysregulated between our patients with LS and those without LS. [file 1246129.f1.zip › supplementary material_3.pdf]

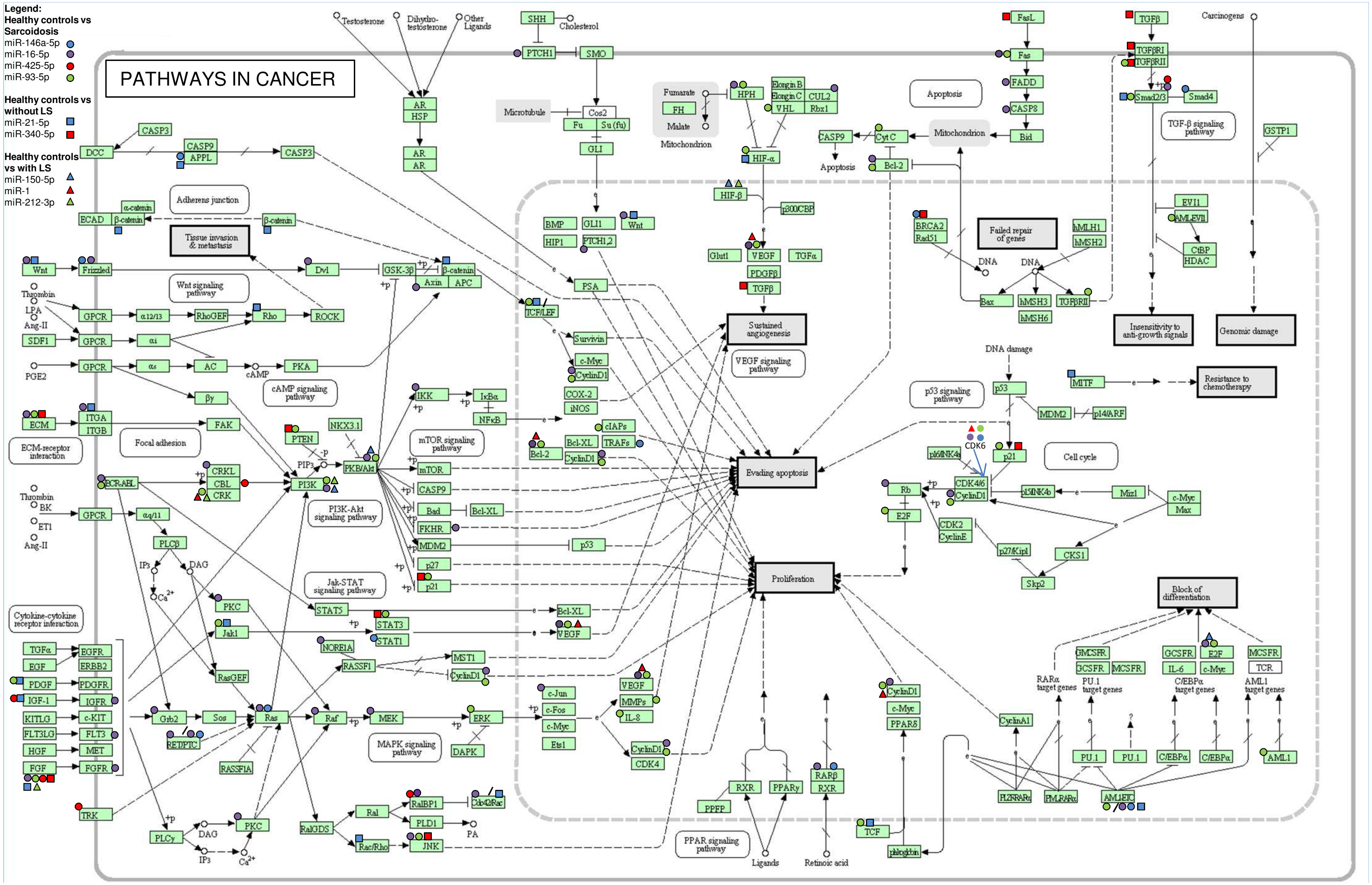

Online Resource 3: A, the Pathways in cancer with its highest MiRSystem score among KEGG pathways was consistently predicted to be affected by both expression profiles that were dysregulated in our patients with/without Löfgren's syndrome (LS) compared to healthy controls; a circle, 4 miRNAs (miR-146a-5p, miR-16-5p, miR-425-5p, miR-93-5p) were consistently dysregulated in both sarcoidosis groups compared to healthy controls; a square: 2 (miR-340-5p, miR-21-5p) miRNAs were only dysregulated in sarcoidosis with LS compared to healthy controls; a triangle, 3 miRNAs (miR-150-5p, miR-1, miR-212-3p) were only dysregulated in sarcoidosis without LS compared to healthy controls

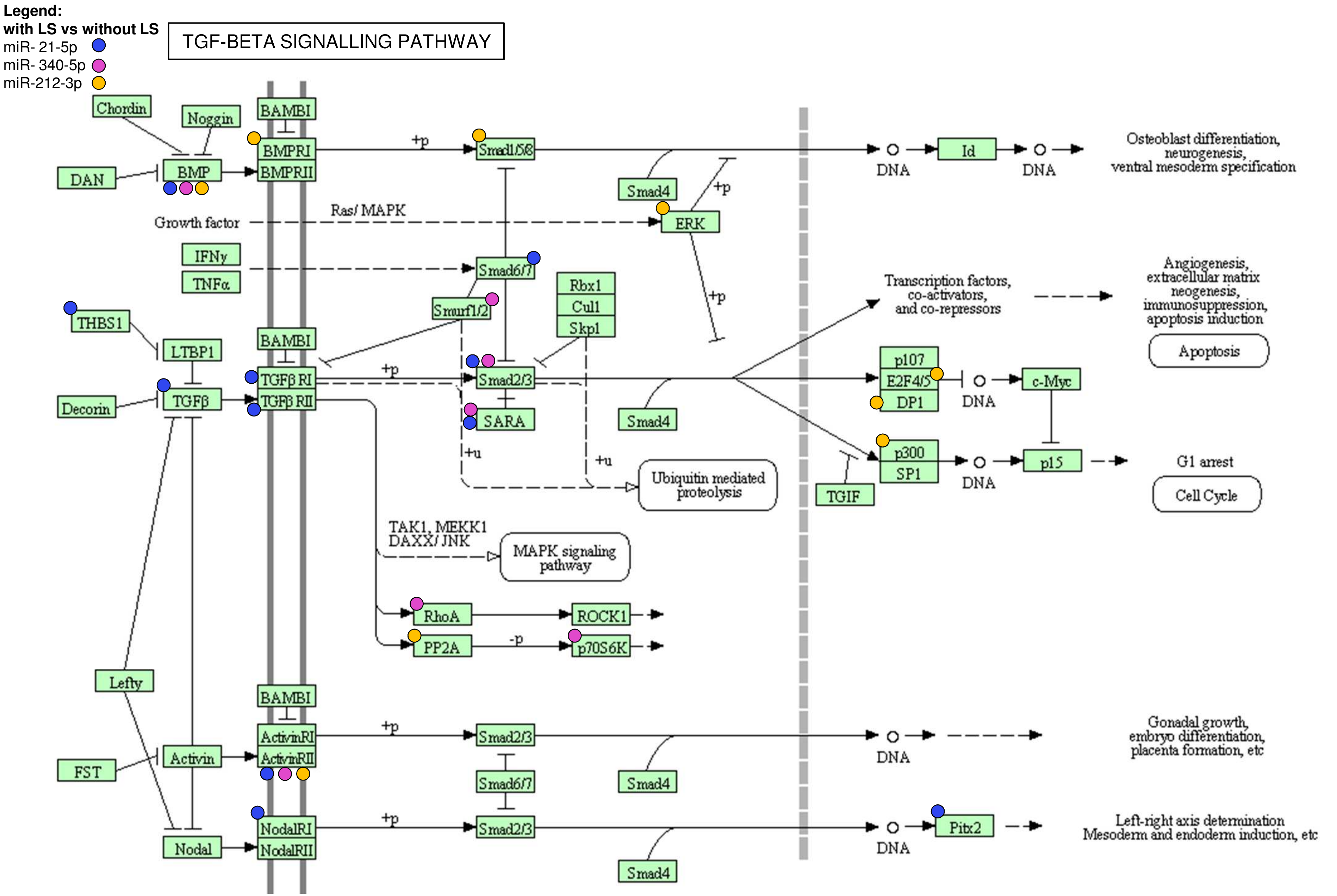

Online Resource 3: B, the TGF-beta signalling pathway with its highest MiRSystem score among KEGG pathways was predicted to be affected by the cumulative effect of three miRNAs whose serum expressions were dysregulated between our patients with Löfgren‘s syndrome and those without Löfgren‘s syndrome; 3 miRNAs (miR-340-5p, miR-212-3p and miR-21-5p) were only dysregulated in sarcoidosis with LS compared to that without LS
